# Average Links Between Daily Gender Expression and Depressive Symptoms Do Not Describe Individual Adolescents

**DOI:** 10.1007/s10964-025-02184-x

**Published:** 2025-04-15

**Authors:** Ran Yan, Christel M. Portengen, Natasha Chaku, Adriene M. Beltz

**Affiliations:** 1 Department of Psychology, University of Michigan, Ann Arbor, MI USA; 2 Department of Clinical Child and Family Studies, Utrecht University, Utrecht, the Netherlands; 3 Department of Psychological and Brain Sciences, Indiana University-Bloomington, Bloomington, IN USA

**Keywords:** Depression, Femininity, Idiographic, Intensive longitudinal study, Masculinity, Person-specific

## Abstract

Gender expression is important for mental health, with masculinity and femininity having differential significance for unique adolescents. Yet, most empirical work on gender expression assumes it is trait-like or similarly shifting across teens. This intensive longitudinal study examined state-like aspects of gender expression and heterogeneity in adolescent-specific associations with depressive symptoms over 100 days. Participants were 106 adolescents, including 5 gender-expansive youth (54.7% cisgirls, 74.5% White; *M*_age_ = 13.31, *SD*_age_ = 1.94). A sample-average link between daily masculinity and reduced symptoms was found for cisboys. Adolescent-specific results qualified this effect: Only ~25% evidenced an association between daily gender-congruent expression—masculinity for cisboys and femininity for cisgirls—and daily reduced symptoms. Using 9000+ daily reports, findings highlight the dynamic nature of gender expression and the need to use a person-specific approach in understanding the heterogenous psychological correlates of masculinity and femininity for today’s youth.

## Introduction

Adolescence is a sensitive developmental period marked by physiological, psychological, and social changes that can increase vulnerability to psychopathology (Pfeifer & Allen, 2021). Gender significantly shapes these experiences (Zahn-Waxler et al., 2008). Diagnoses of depressive disorders nearly double from ages 13 to 17, evidencing large gender differences that persist into adulthood (Albert, 2015; Merikangas et al., 2010). Indeed, adolescent girls are more likely than boys to develop severe, recurrent symptoms and receive depression diagnoses (e.g., Salk et al., 2017). Comparatively, adolescent boys are less likely to seek help for mental health problems (Sheikh et al., 2025). Although these average gender differences have been well-established in prior research, the unique and fluid ways in which adolescents’ gendered experiences relate to their daily mental health remain underexplored. Therefore, this study examines the daily fluctuations in adolescents’ gendered experiences and person-specific daily links between their gender expression and depressive symptoms over 100 days.

### Gender Expression in Adolescence

Gender is wonderfully complex and multidimensional, and adolescence is critical for gender identity development and gender expression (Steensma et al., 2013). Gender identity is the multifaceted sense of one’s gender (and other genders), encompassing thoughts and feelings about one’s assigned sex at birth (Egan & Perry, 2001). Traditionally viewed as binary, gender identity is now increasingly recognized as a spectrum, including cisgender boy/man and girl/woman identities, as well as gender-nonconforming, non-binary, gender-fluid, transgender, and other gender-expansive identities. Gender-expansive reflects identities and expressions that do not align with traditional expectations for one’s sex assigned at birth, meaning that they lie between, expand beyond, or do not follow gender spectra (Saltis et al., 2023). According to the gender intensification hypothesis, gender identity becomes increasingly congruent and solidified in adolescence, presumably leading to stable gender self-concepts and increased differentiation between boys and girls (Hill & Lynch, 1983). However, empirical evidence for gender intensification is mixed, with some studies reporting evidence for intensification of gender self-concepts in early to mid-adolescence (e.g., Klaczynski et al., 2020), but most studies reporting null effects (e.g., Block et al., 2018). Discrepancies might be especially relevant to modern youth, with current recognition that adolescents experience gender in unique ways.

Gender expression refers to how someone acts in the perceived context of their gender identity, such as feminine attire or masculine behavior (Egan & Perry, 2001). Approximately 25% of adolescents have incongruent gender expressions (i.e., gender expression that does not seemingly align with their gender identity), with cisgender girls having more fluid gender expressions than boys (Becker et al., 2017). Adolescents with incongruent expressions have often reported lower self-worth and self-esteem, and more behavior problems compared to those with congruent expressions (DiDonato & Berenbaum, 2011; Yunger et al., 2004). This may partly result from perceived non-normativity and social backlash of gender-norm violations, especially for cisgender boys (Roberts et al., 2013). Yet, the social context of adolescent gender development is ever-changing: There is a paradoxical rise in the monitoring and legislating of gender and gender-related behaviors in the United States that is simultaneously occurring with increased personal and public acceptance of gender-expansive identities and expressions (Redfield et al., 2024). Thus, there exists a significant knowledge gap concerning the relation between gender expression and mental health in today’s diverse youth.

### Daily Variability in Gender Expression

Filling that knowledge gap requires empirical consideration of variation in gender expression. Variations can occur over time and across individuals. Regarding time, gender expression is conceptualized as a stable, trait-like attribute in most studies (e.g., Potter et al., 2020), but gender expression is unlikely static. It likely fluctuates over relatively short periods of time, with shifts influenced by distal or proximal factors such as verbal comments (e.g., about stereotypically gendered clothing), visual stimuli (e.g., sexualized media), and gender of interaction partners (e.g., “power poses” in a meeting; Leszczynski & Strough, 2008; Mehta & Dementieva, 2017). Indeed, two intensive longitudinal studies showed that 88–93% of cisgender adults experienced daily fluctuations in feminine and masculine expression, and more so among women than men during young adulthood (Beltz et al., 2021; Yan et al., 2024).

Regarding individuality, most studies concern “average” cisgender adolescents, using between-person analytic approaches for cross-sectional data with results that reflect variation across people (i.e., nomothetic research). Although a reasonable starting point, nomothetic research is an unreasonable end point, as adolescents increasingly endorse non-cisgender identities, and as great variability exists within groups of cisgender teens (Becker et al., 2017). Consequently, the mathematical assumptions required for nomothetic research (i.e., homogeneity over time and across people) are likely violated (Molenaar, 2004). To understand the ‘individual’ adolescent, whose gender is uniquely expressed, experienced, and lived, idiographic research is imperative. It leverages intensive longitudinal designs and within-person analytic approaches for data collected at frequent intervals with results that reflect a sample of N = 1 (Beltz et al., 2016).

Idiographic research holds great promise for capturing the heterogeneity of adolescent gender and mental health, as evidenced by a 75-day study in young adults (Beltz et al., 2021). Results revealed a meaningful link between gender expression and depressive symptoms for the majority of the cisgender sample, and the direction of the link often aligned with the gender congruence effect; days with reduced depressive symptoms co-occurred with heightened masculine expressions for men and heightened feminine expressions for women. This effect was more common in men (42%) than women (27%) (Beltz et al., 2021). About 40% of the sample, though, showed no link between daily gender expression and depressive symptoms, and a minority even reported increased depressive symptoms on congruent days. Similar but more pronounced results were seen in a study focused on depressive symptoms in cisgender middle adults, with >50% of women and >35% of men showing a gender congruence effect, and with no men evidencing a link in the opposite direction (i.e., reduced daily masculinity relating to reduced daily depressive symptoms; Yan et al., 2024). Importantly, the strength and direction of these daily correlations were unrelated to average levels of depressive symptoms in this sample (Yan et al., 2024); in other words, those with higher baseline depression were not more or less likely to have symptoms that varied in relation to gender expression. These studies highlight the value of an idiographic approach for studying gender, gender expression, and depression, but it remains unclear if and how their results extend to adolescence.

## Current Study

Given the importance of identity formation in adolescence, a period of developmental transitions in internalizing problems and gendered daily experiences, the current study aims were to examine fluctuations in daily gender expression (aim 1), and to examine heterogeneity in adolescent-specific associations between gender expression and depressive symptoms over 100 days (aim 2). Regarding the first aim, at the sample-level, gender expression and depression were expected to fluctuate, with cisgender girls showing more variation than boys. A gender congruence effect was also expected (i.e., fewer symptoms on days cisboys felt more masculine but cisgirls felt more feminine), especially among cisboys. Regarding the second aim, at the individual-level, heterogeneity in person-specific correlations between gender expression and depressive symptoms (i.e., directionality and magnitude) was expected, highlighting the uniqueness of the daily relations between depression and gender expression for adolescents, especially among cisgirls. Individual differences in daily correlations were not expected to be related to average depressive symptoms, as symptom fluctuations with gender expression – though meaningful for individuals – do not reflect overall mental health.

## Methods

Data were collected online between March 2021 and August 2022 as part of larger study on adolescent daily experiences conducted by a research laboratory in the United States. The study protocol was approved by the Institutional Research Board at the University of Michigan. All procedures comply with the ethical standards of the relevant national and institutional committees on human experimentation and with the Helsinki Declaration of 1975, as revised in 2008. Data on inhibitory control and externalizing behaviors were previously reported (Chaku et al., 2024).

### Participants

The final sample consisted of 106 adolescents (43 cisgender boys, 58 cisgender girls, 5 gender-expansive^1^) aged 9–18 years (*M* = 13.31, *SD* = 1.93) with an average daily completion rate of 94.3%. Adolescents reported their gender identity: boy, girl, transgender, non-binary, gender-fluid, or open-ended response. Cisgender and transgender identities were classified by comparing adolescents’ gender identity response with their sex assigned at birth. The sample consisted of predominantly White (74.5%) and non-Latine (91.5%) youth, with others identifying as Black/African American (6.6%), American Indian/Alaskan Native (1.0%), and more than one race (17.9%); it consisted of 44 sibling pairs and 18 singletons. Cisgender boys and girls were comparable in age, *t*(99) = −1.84, *p* = 0.069, race, *χ²*(3) = 1.43, *p* = 0.699, and ethnicity, *χ²*(1) = 1.41, *p* = 0.235. Thirteen adolescents (7 cisgender girls and 2 gender-expansive youth) were at risk for clinical depression according to a baseline measurement with established criteria (i.e., a Center for Epidemiologic Studies Depression Scale sum score ≥16; Radloff, 1977).

The sample originated from 139 adolescents (58 cisgender boys, 75 cisgender girls, 6 gender-expansive) who completed an intensive longitudinal study in which they filled out questionnaires for 100 consecutive days. Families consisting of one parent or legal guardian and two children between the ages of 8 and 21 years (one of which had to be aged 8 to 17 years) were recruited through social media, virtual flyers, and university-affiliated online databases. The sample excluded adolescents who completed fewer than 80% of the daily surveys (*n* = 27) to ensure data fidelity, following previous intensive longitudinal research and simulations showing that <20% missingness does not impact inferences (Rankin & Marsh, 1985; Wright et al., 2019). Included and excluded adolescents were comparable in age, *t*(131) = −1.69, *p* = 0.093, gender, *χ²*(2) = 0.20, *p* = 0.907, race, *χ²*(3) = 1.51, *p* = 0.681, and ethnicity, *χ²*(1) = 0.18, *p* = 0.671. They also did not significantly differ in mean daily depressive symptoms (i*M*, described below), *t*(131) = −1.89, *p* = 0.061.

### Procedure

During an online intake session, parents completed an electronic informed consent form for themselves and their participating child(ren) under the age of 18, who provided additional informed assent. Adolescents aged ≥18 years provided informed consent. Parents and adolescents then completed online surveys via Qualtrics.

The following evening, adolescents began daily surveys, which are the focus of this report. Specifically, 20-min surveys were sent to parents’ email addresses (for adolescents aged <18 years) or adolescents’ email addresses (if aged ≥18 years) each day at 5:00PM for 100 days. Adolescents were instructed to take the survey at 8:00PM or after they finished that day’s activities. Families received $15 per member for completing the intake survey. Adolescents received $1 for each daily survey they completed, which doubled if adolescents completed ≥80% of the surveys. Adolescents received an additional $35 bonus if they completed ≥90% of the surveys. Participants were withdrawn from the study if they did not fill out at least 15 diaries in the first 30 days, or their completion rate dropped below 50% any day thereafter.

### Daily Measures

#### Daily Depressive Symptoms

The Short Mood and Feelings Questionnaire (SMFQ) was adapted to assess adolescents’ depressive symptoms in the past 24 hours (Messer et al., 1995). Adolescents were asked to rate 13 items regarding their thoughts and feelings that day (e.g., “I didn’t enjoy anything at all”) on a three-point Likert scale from 0 (*not true*) to 2 (*true*). A composite score was calculated per day by averaging across items, with higher scores indicating elevated symptoms. The SMFQ is a valid and reliable instrument for assessing depressive symptoms in general and particularly in adolescents, and the daily version implemented here was reliable: Based on multilevel confirmatory factor analysis (Geldhof et al., 2014), the measure had excellent internal consistency between and within participants (Ω_between _= 0.958; Ω_within_ = 0.843) in this sample.

#### Daily Gender Expression

The Sex Role Identity Scale (Storms, 1979) was adapted to measure daily self-perceived masculinity and femininity in the past 24 hours. Participants rated 6 items regarding how masculine and then how feminine: they acted, appeared or came across that day; their personality was that day; and they thought they were in general that day on a 5-point Likert scale from 1 (*not at all*) to 5 (*extremely*). The terms masculine and feminine were not defined. The three femininity items were reverse-coded and combined with the three masculinity items to create a feminine-to-masculine continuum composite score for each day, following previous studies (Beltz et al., 2021). Lower scores indicate self-perceived femininity, and higher scores self-perceived masculinity. The measure is internally valid and reliable in this sample, with the daily version demonstrating excellent between-person and good within-person reliability (Ω_between _= 0.968; Ω_within_ = 0.711).

Daily average scores were also calculated separately for masculinity (Ω_between _= 0.998; Ω_within_ = 0.863) and femininity (Ω_between _= 0.997; Ω_within_ = 0.866), considering theories conceptualizing them as separate constructs (e.g., Antill et al., 1993). Higher scores indicate greater masculinity and femininity, respectively.

### Data Analysis Plan

Three sets of analyses were conducted focusing on gender expression as a continuum (with secondary analyses examining masculinity and femininity separately). First, the intraindividual mean (i*M*) and intraindividual standard deviation (i*SD*) across the 100 days for each adolescent’s gender expression composites were calculated. Each i*SD* reflects variation from each adolescent’s daily mean (i*M)*. One-sample *t*-tests examined whether i*SD*s differed from zero, indicating significant fluctuations. Finally, i*M*s and i*SD*s for gender expression were compared between cisgender boys and girls using two-sample *t*-tests^2^, and the i*SD*s were correlated with age and average daily depressive symptoms. Multilevel models, adjusting for sibling dependencies, were also conducted for between-person comparisons.

Second, multilevel models using maximum likelihood estimation with unstructured error covariance were conducted in IBM SPSS Statistics (version 29) to examine the psychological impact of daily gender expression in cisgender adolescents at the sample-level. Daily depressive symptoms were predicted by gender expression (grand mean-centered), gender (boys coded 0), and their interaction, accounting for individual-level (daily) and family-level (sibling) dependencies, and with age as a covariate due to its range within the sample. The sample size was sufficient to detect a small interactions between gender and gender expression (Cohen’s *f* = 0.06; Faul et al., 2007), with 80 repeated observations (correlated ~0.3), 80% power, and *α* = 0.05.

Third, daily links between gender expression and depressive symptoms were examined in all adolescents who evidenced fluctuations in their gender expression and depressive symptoms (i*SD*s ≠ 0), using person-specific correlations in R (version 4.2.1). For each adolescent, gender expression and depressive symptoms daily composites were predicted by their own scores from the previous day, and residuals were used in correlations with each other (see Heath, 2014). The absolute values of the correlations were linked to age and average daily depressive symptoms. These residualized correlations were then used to index whether the daily synchrony between gender expression and depressive symptoms was meaningful for an adolescent, that is, whether the correlation exceeded the smallest effect size of interest (SESOI) at *r* = |0.10| (Cohen, 1988). Finally, for cisgender adolescents, the correlations indicated whether the relation evidenced a gender congruence effect (i.e., negative correlations reflecting increased masculinity [high continuum scores] and reduced symptoms for boys, or positive correlations reflecting increased femininity [low continuum scores] and reduced symptoms for girls). To explore whether gender congruence was linked to age or mattered for overall depressive symptoms, cisgender adolescents who did and did not evidence a gender congruence effect were then compared with respect to their age and average daily depressive symptoms (i*M*s).

## Results

### Fluctuations in Daily Gender Expression and Depressive Symptoms across 100 Days

The average gender expression score (i*M*) across the full sample (*N* = 106) was around the midpoint of the scale (*M* = 2.92, *SD* = 1.40). The average intraindividual standard deviation (i*SD*) of 0.26 (*SD* = 0.18) was significantly different from zero, *t*(105) = 15.43, *p* < 0.001, *d* = 1.50, indicating meaningful fluctuations in feminine-to-masculine expression over 100 days. Gender expression i*SD*s ranged from 0 to 0.87 across participants, with 92.5% of adolescents showing fluctuations. Eight adolescents (4 cisboys and 4 cisgirls) showed absolute stability in their gender expression (i.e., i*SD* = 0).^3^

The average masculinity score was 2.76 (*SD* = 1.47) and showed significant daily fluctuations (*M*_i*SD=*_0.32, *SD* = 0.24) across 100 days, *t*(105) = 13.62, *p* < 0.001, with i*SD*s ranging from 0 to 0.93 and 87.7% of individuals reporting fluctuations. Thirteen individuals (5 cisboys and 8 cisgirls) reported absolute stability. Similarly, the average femininity score was 2.91 (*SD* = 1.45) and showed significant fluctuations (*M*_*iSD*_ = 0.34, *SD* = 0.27) across days, *t*(105) = 13.23, *p* < 0.001, with i*SD*s ranging from 0 to 1.16, and 84.9% reported fluctuations. Sixteen individuals (9 cisboys and 7 cisgirls) reported absolute stability.

Daily gender expression (i*M*s) significantly differed between cisgender boys and girls, with a very large effect size, *t*(99) = 16.98, *p* < 0.001, *d* = 3.41. Cisboys reported average self-perceptions in the masculine range (*M* = 4.31, *SD* = 0.60; range: 3.03–5.00), and cisgirls averaged in the feminine range (*M* = 1.87, *SD* = 0.79; range: 1.00–3.94) of the continuum. Cisgender boys (*M* = 0.25, *SD* = 0.16) and girls (*M* = 0.26, *SD* = 0.17) showed comparable fluctuations in daily gender expression, *t*(99) = −0.18, *p* = 0.855, *d* = 0.04. There was a significant association between fluctuations (i*SD*s) in daily gender expression and average daily depressive symptoms (i*M*), *r*(99) = 0.22, *p* = 0.030, but not age, *r*(99) = −0.10, *p* = 0.331. The pattern of results was the same in multilevel models accounting for family dependencies, presented in Supplementary Material.

Consistent with the continuum results, average masculinity was higher for cisgender boys (*M* = 4.10, *SD* = 1.01) than girls (*M* = 1.72, *SD* = 0.83), *t*(99) = 12.98, *p* < 0.001, *d* = 2.60, and average femininity was higher for cisgender girls (*M* = 3.99, *SD* = 0.89) than boys (*M* = 1.48, *SD* = 0.54), *t*(99) = −16.41, *p* < 0.001, *d* = −3.30. Cisgender boys (*M*_*iSD*_ = 0.33, *SD* = 0.24) and girls (*M*_*iSD*_ = 0.30, *SD* = 0.24) showed comparable daily masculinity fluctuations, *t*(99) = 0.79, *p* = 0.431, *d* = 0.18, and daily femininity fluctuations (cisboys: *M*_*iSD*_ = 0.31, *SD* = 0.28, cisgirls: *M*_*iSD*_ = 0.36, *SD* = 0.25), *t*(99) = −0.89, *p* = 0.378, *d* = 0.18. There were no significant associations between fluctuations in daily masculinity or femininity and age, *r*(99) = −0.19, *p* = 0.054 and *r*(99) = −0.04, *p* = 0.683, respectively. The pattern of results was the same in multilevel models, presented in Supplementary Material, except that the relation between masculinity and age was significant when correcting for family dependencies.

### Sample-level Links between Daily Gender Expression and Depressive Symptoms

Multilevel models revealed a significant interaction between gender and gender expression (*p* = 0.023), indicating that the daily relation between femininity-to-masculinity and depressive symptoms differed for cisgender boys and girls; see Table 1. Follow-up models were run separately for cisgender boys and girls, with results showing that gender expression was significantly and inversely associated with depressive symptoms for cisboys (*p* = 0.010) but not cisgirls (*p* = 0.524). Specifically, cisboys reported lower depressive symptoms on days they felt more masculine, evidencing an average gender congruence effect. Age was not a significant covariate in any model, but significant random effects for the intercepts and gender expression slopes highlight family and individual variability around the estimates.^4^

**Table 1:** Multilevel model predicting depressive symptoms by cisgender and gender expression continuum, with an age covariate

	Full sample (*N* = 101)			Cisgender boys (*N* = 43)			Cisgender girls (*N* = 58)		
Fixed effects	*b*	*SE*	*p*	*b*	*SE*	*p*	*b*	*SE*	*p*
Intercept	0.03	0.06	0.556	0.09	0.03	0.003	0.18	0.07	0.015
Age	0.02	0.01	0.183	0.002	0.003	0.430	−0.002	0.01	0.873
Gender (0 = cisboys)	0.09	0.05	0.079	__	__	__	__	__	__
Expression	**−0.04**	**0.02**	**0.043**	**−0.04**	**0.01**	**0.010**	0.01	0.02	0.524
Gender X Expression	**0.05**	**0.02**	**0.023**	__	__	__	__	__	__
**Random effects**									
Intercept	0.05	0.01	<0.001	0.02	0.006	<0.001	0.08	0.02	<0.001
Expression	0.01	0.002	<0.001	0.01	0.004	<0.001	0.01	0.004	0.009
**Model fit**									
AIC	−6292.06			−6023.62			−1775.12		

Statistically significant fixed effects are in bold, and the gender expression continuum ranged from femininity (low scores) to masculinity (high scores)

Secondary multilevel models were run using masculinity and femininity as separate predictors. For masculinity, there was a significant interaction with gender on depressive symptoms (*p* = 0.016); see Supplementary Material, Table S2. Follow-up models indicated that masculinity was significantly inversely linked to depressive symptoms for cisboys (*p* = 0.006) but not cisgirls (*p* = 0.944). For femininity, there was no significant interaction with gender (*p* = 0.254), but gender was a significant predictor of depressive symptoms (*p* = 0.003); see Supplementary Material, Table S3. Cisgender girls, on average, reported higher levels of symptoms than cisgender boys, as expected. For both models, age was not a significant covariate, but random effects for the intercepts as well as masculinity and femininity slopes were significant.

### Individual-level Links between Daily Gender Expression and Depressive Symptoms in Cisgender Adolescents

Person-specific correlations between gender expression and depressive symptoms in cisgender adolescents (*n* = 90) ranged from *r* = −0.77 to *r* = 0.60. Despite the sample-average correlation being ~0 (*M* = 0.003, *SD* = 0.23), 46.7% of adolescents had correlations that exceeded the SESOI (*r* > |0.1|); this reflected 43.2% of cisboys (range: −0.77–0.60, *M* = −0.04, *SD* = 0.60) and 49.1% of cisgirls (range: −0.55–0.44, *M* = 0.03, *SD* = 0.17). There was no significant association between the strength (i.e., absolute value) of the correlation and average daily depressive symptoms (i*M*s), *r*(88) = 0.09, *p* = 0.385, or age, *r*(88) = 0.12, *p* = 0.249.

**Fig. 1:**
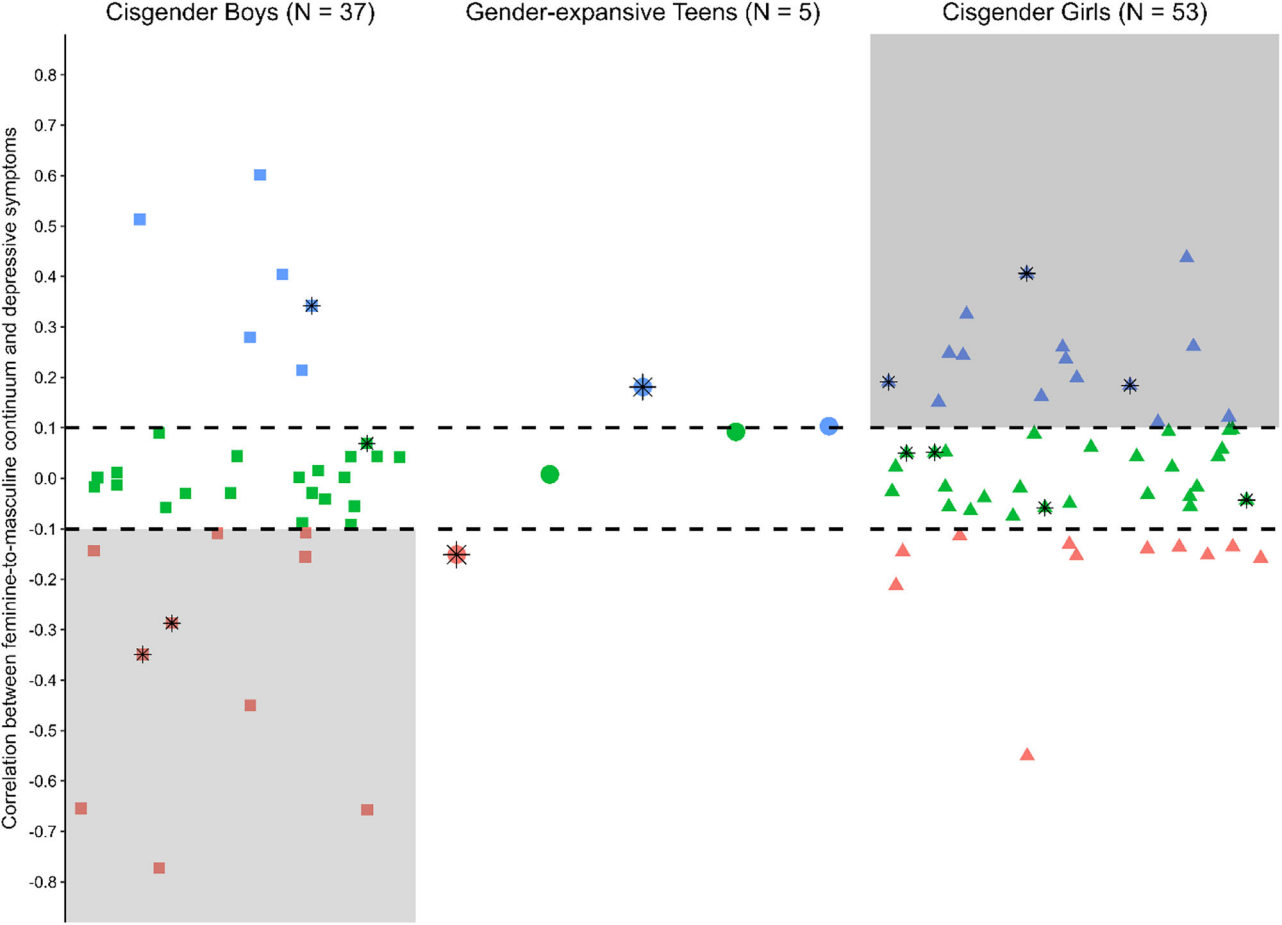
Scatterplot displaying adolescent-specific correlations between daily gender expression (measured on a feminine-to-masculine continuum) and depressive symptoms among 95 adolescents. The y-axis shows the strength and direction of the correlations, and the x-axis represents individual adolescents. Cisgender boys are indicated by squares on the left side of the plot, gender-expansive teens are indicated by circles in the middle, and cisgender girls are indicated by triangles on the right. Youth at risk for clinical depression (*n* = 13) are overlaid with a black asterisk. The thick dashed lines at *r* = 0.10 and *r* = −0.10 demarcate the threshold for the smallest effect size of interest. Positive correlations, (shown in blue above the dashed lines) and negative correlations (shown in red below the dashed lines) signify meaningful individual-level correlations. The gray shaded areas highlight gender congruence effects

The left and right sides of Fig. 1 display these relations. Each point corresponds to a 100-day correlation between gender expression and depressive symptoms for an adolescent (cisboys: squares; cisgirls: triangles), with youth at risk for clinical depression indicated with asterisks. The dashed lines mark the threshold for the SESOI, and strikingly 56.8% of cisboys and 50.9% of cisgirls did not show a meaningful link between gender expression and depressive symptoms (i.e., scores located in between the dashed lines). The shaded areas in the figure represent gender congruence effects, where for cisboys, higher femininity-to-masculinity expression scores are associated with reduced depressive symptoms, and for cisgirls, higher scores are associated with increased symptoms. For 27.0% of cisboys and 28.3% of cisgirls, gender congruence effects were found. The remaining adolescents (16.2% of cisboys and 20.8% of cisgirls) actually showed the reverse (gender incongruence) pattern. Lastly, youth at risk for clinical depression did not evidence clear patterns in the scatterplot, providing further evidence that average depression levels were not associated with daily links between gender expression and depressive symptoms.

**Fig. 2:**
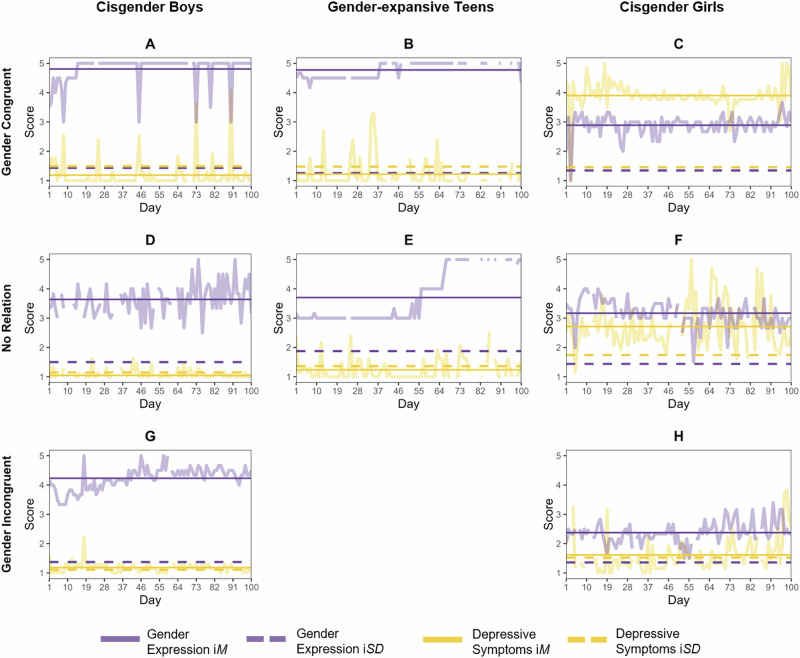
Plots of daily gender expression and depressive symptoms for illustrative adolescents in separate panels (**A**−**H**) over 100 study days, with study day on the x-axis and feminine-to-masculine expression scores (purple) and depressive symptom scores scaled to 1–5 (yellow) on the y-axis. Plots show some cisgender boys (left; panel **A**, **D** and **G**), gender-expansive youth (middle; panel **B** and **E**), and cisgender girls (right; panel **C**, **F** and **H**) as well gender congruent patterns (top; panel **A**, **B** and **C**), no meaningful relations (middle; panel **D**, **E** and **F**), and gender incongruent patterns (bottom; panel **G** and **H**). Solid horizontal lines represent average scores across 100 days (i*M*), and dashed horizontal lines represent intra-individual standard deviations (i*SD*) plus 1 (to facilitate visualization on the 1–5 scale)

Plots of daily gender expression and depressive symptoms for illustrative individuals are shown in Fig. 2, with cisgender youth on the left and right sides. Specifically, participants A (cisgender boy) and C (cisgender girl) exhibited gender-congruence effects. Depressive symptoms (in yellow) increased on days when feminine-to-masculine expression (in purple) decreased for participant A but increased for participant C. In contrast, participants G (cisgender boy) and H (cisgender girl) displayed gender-incongruence effects. Notice, for instance, the day 18 spike in both depressive symptoms and masculinity for participant G. Finally, no meaningful correlations were found for participants D (cisgender boy) and F (cisgender girl), evidenced by the lack of discernible patterns within the rises and falls of daily symptoms and expression.

A crucial question concerns whether gender congruence mattered for overall levels of adolescents’ depressive symptoms across the study or characteristics linked to gender development (i.e., age). There was no significant difference in the symptom i*M*s of adolescents who evidenced a gender congruence effect (*n* = 25; *M* = 0.16, *SD* = 0.30) and those who did not (*n* = 17; *M* = 0.10, *SD* = 0.12), *t*(40) = 0.79, *p* = 0.434, *d* = 0.25. There was also no significant difference in age between those who evidenced a gender congruence effect (*M* = 13.63, *SD* = 2.16) and those who did not (*M* = 13.76, *SD* = 1.49), *t*(40) = −0.20, *p* = 0.840. To account for family dependencies, analyses were repeated with only one sibling per family; the sibling with an age closest to the sample mean was retained (excluding 3 cisboys and 2 cisgirls). The same pattern of results was found; see the Supplementary Material.

Secondary analyses in cisgender adolescents used masculinity and femininity separately in person-specific correlations with daily depressive symptoms. For masculinity (*n* = 86)^3^, 43% of correlations exceeded the SESOI. This reflected 40.5% of cisboys (range: −0.89–0.46, *M* = −0.08, *SD* = 0.28) and 44.9% of cisgirls (range: −0.32–0.44, *M* = 0.04, *SD* = 0.16), with no significant difference per cisgender group, *χ*^2^(1) = 0.03, *p* = 0.854, or association with age, *r*(84) = 0.12, *p* = 0.270. Specifically, gender congruent expression (i.e., cisboys feeling more masculine and cisgirls feeling less masculine) was linked to fewer depressive symptoms in 27.0% of cisgender boys and 32.7% of cisgender girls, with no meaningful daily association observed for 59.5% of cisgender boys and 55.1% of cisgender girls. Notably, 13.5% of cisgender boys and 12.2% of cisgender girls evidenced a gender incongruent pattern; see Supplementary Material, Fig. S1.

For femininity (*n* = 83)^3^, 47% of correlations exceeded the SESOI. This reflected 39.4% of cisgender boys (range: −0.47–0.66, *M* = −0.02, *SD* = 0.23) and 52.0% of cisgender girls (range: −0.32–0.61, *M* = −0.02, *SD* = 0.17), with no significant difference by gender, *χ*^2^(1) = 0.81, *p* = 0.367, or association with age, *r*(81) = −0.03, *p* = 0.326. Gender congruent expression (i.e., cisboys feeling less feminine and cisgirls feeling more feminine) was associated with reduced depressive symptoms in 18.2% of cisgender boys and 26% of cisgender girls, with no meaningful daily association observed for 60.6% of cisgender boys and 48.0% of cisgender girls, and with 21.2% of cisgender boys and 26.0% of cisgender girls exhibiting a gender incongruent pattern; Supplementary Material, Fig. S2.

### Individual-level Links between Daily Gender Expression and Depressive Symptoms in Gender-expansive Adolescents

Information regarding fluctuations in daily gender expression for the 5 gender-expansive youth are described in the Supplementary Material. Person-specific correlations between gender expression and daily depressive symptoms for gender-expansive adolescents are shown as circles in the middle of Fig. 1 (range: −0.15–0.18). One individual evidenced a negative correlation (red circle), whereas two others showed a positive correlation (blue circles). Importantly, there are two individuals (green circles) for whom daily gender expression was not meaningfully linked to depressive symptoms (i.e., correlations that did not exceed the SESOI).

The person-specificity of gender expression and its relation with depressive symptoms is highlighted for two adolescents in the middle of Fig. 2. Participant B exhibited an inverse relation between feminine-to-masculine expression and depressive symptoms, with expression in the masculine range every day (i.e., ≥4). Participant E, however, evidenced no meaningful link between gender expression and symptoms.

Secondary person-specific analyses examining masculinity and femininity separately in gender-expansive teens revealed results consistent with these patterns. For masculinity (Fig. S1), adolescent-specific correlations with daily depressive symptoms ranged from −0.30 to 0.14, with 3 participants showing no meaningful association. For femininity (Fig. S2), correlations ranged from −0.31 to 0.16, with 2 adolescents evidencing no meaningful links.

## Discussion

Gender differences in depressive symptoms first emerge in adolescence but there is little consensus on what role daily gender expressions play in adolescent mental health. Using 100-day intensive longitudinal data from over 100 cisgender and gender-expansive adolescents – reflecting over 9000 daily reports – the current study highlights daily fluctuations in how youth express their gender and the person-specific ways in which these expressions relate to daily mental health (i.e., depressive symptoms). As hypothesized, gender expression showed significant variability from each person’s mean across 100 days. Moreover, the role that gender expression played in youth daily depressive symptoms showed considerable heterogeneity, despite mean-level findings indicating that fluctuations were positively related to daily depressive symptoms, and that daily gender expression, and especially masculinity, was negatively related to daily depressive symptoms in cisgender boys.

### Interpretations and Implications

As expected, the vast majority (92%) of adolescents showed notable variation in gender expression across study days, consistent with previous research noting the state-like and contextual nature of gender expression (e.g., Beltz et al., 2021; Mehta & Dementieva, 2017). This was true when gender expression was conceptualized as a feminine-to-masculine continuum or when femininity and masculinity were considered as distinct dimensions. Although past work has shown greater fluctuations in adult women than men (Beltz et al., 2021) and greater gender rigidity in adolescent boys than girls (Nielson et al., 2024), this study found no gender differences in fluctuations in daily gender expression. This may be because public opinions on gender non-normativity are more tolerant among today’s youth (Allen et al., 2022), potentially signaling changing gender norms for cisgender boys. Such trends could be related to the measure of gender expression used in this study, which is based on adolescents’ own perceptions of masculinity and femininity (e.g., aspects of appearance considered maximally “masculine” in 2024 and 1994 likely differ).

At the sample-level, daily fluctuations in gender expression were related to daily fluctuations in depressive symptoms among cisgender adolescents, but only for cisboys such that days with higher masculinity co-occurred with reduced depressive symptoms. Similar gender differences have been reported in young adults (Beltz et al., 2021). The role of masculinity in cisgender boys’ mental health is well-documented, with threats to boys’ masculinity strongly affecting their mental health (Exner-Cortens et al., 2021). Given that adolescent (cisgender) girls still have higher levels of daily depressive symptoms than boys (in this and other studies; e.g., Shorey et al., 2022), it seems unlikely that gender expression is responsible for the widely-reported gender disparity in depression, but instead it might reflect a specific aspect of depression for boys and men.

The results of this study also indicate that the daily relation between gender expression and depressive symptoms is not just sex-specific, but also specific to each adolescent, with changes occurring regardless of age. This is evident in daily correlations for individual adolescent cisgender boys, as it was expected that a majority would show a gender congruence effect (i.e., inverse relation between daily masculinity and depression, consistent with the sample-level results and past work with young adults; Beltz et al., 2021). Only 27%, however, demonstrated that relation. Adolescence is a broad developmental period of identity exploration, possibly leading to less social backlash for gender non-conformity compared to young adulthood. Moreover, reduced stigma and increased support for diverse gender identities in today’s generation may downplay the mental health impact of gender non-conformity in some contexts (Olson et al., 2016). Consistent with this, the majority of the sample – representing a wide range of adolescent ages – did not have a meaningful daily link between gender expression and depressive symptoms, contrasting findings in young and middle adulthood (Beltz et al., 2021; Yan et al., 2024). A minority of cisboys even had a positive relation between masculinity and depressive symptoms, perhaps demonstrating how strict adherence to masculinity norms can negatively affect mental health for some (Leaper et al., 2019).

The adolescent-specificity of gender expression and mental health is also clearly seen in daily relations of cisgender girls and adolescents with expansive identities. Only about a quarter of cisgirls showed a gender congruent link, similar to findings in young adult women (Beltz et al., 2021), but less than the ~50% of women who showed a gender congruent effect in middle adulthood (Yan et al., 2024). Another quarter of cisgender girls showed a gender incongruent link – potentially indicating a group of girls who show more pressure to express themselves in ways that align with their gender to avoid social backlash, particularly as a historically less powerful gender (Javdani, 2012). Adolescent-specific analyses were also essential in understanding the unique experiences of gender and its potential role in mental health for gender-expansive teens. In this study, 3 of 5 gender-expansive youth had meaningful daily links between expression and mental health. Importantly, it was not required to classify these individuals into gender “groups” to make inferences, highlighting the utility of an idiographic approach via intensive longitudinal data for demonstrating heterogeneity in gender expression and psychological adjustment (Diamond, 2020).

### Study Considerations and Future Directions

The current study utilized a 100-day design to ensure statistical power for person-specific inferences between daily gender expression and depressive symptoms, but the subsample of gender-expansive adolescents was small. Studies with larger samples of gender-expansive adolescents are needed. Selection effects for individuals willing to participate in intensive longitudinal research might also be present (e.g., in motivation and inhibitory control; Chaku et al., 2024), but this is not universally seen (Wrzus & Neubauer, 2022), and participants who did and did not complete ≥80% of this study’s days did not differ in demographics or mean level of daily depressive symptoms.

Moreover, this study focused on time-locked, daily relations between gender expression (as a single continuum, and separately as masculine and feminine dimensions) and depressive symptoms (after adjusting for autocorrelations) using sample-level (i.e., multilevel models) and individual-level (i.e., person-specific correlations) analyses. Although over 90% of adolescents reported fluctuations, 8 adolescents who reported no fluctuation in their gender expression across the 100 days could not be included in the person-specific analyses, that is, they reported exactly the same level on the feminine-to-masculine continuum every day. It will be important for future work to understand the nature and context and to reveal the mechanisms underlying these remarkably consistent reports. These youth may express their gender in consistent ways because they do not think about their gender often (e.g., low gender salience; Hinton et al., 2024), because they closely monitor their presentation (e.g., Redfield et al., 2024), or for other reasons. Moreover, the person-specific analyses cannot speak to the directionality of the daily relations between daily gender expression and depressive symptoms; it just reveals the extent to which they rise and fall in synchrony. Future work with time-lagged models could provide insight into whether symptom changes or expression changes drive the relation (Hamaker et al., 2015).

It also remains unclear if daily assessments are an ideal timeframe, or if repeated daily assessments systematically alter how adolescents report on their expression or symptoms. Thus, these findings encourage future work generalizing across timeframes and in the context of a broad network of daily behaviors (e.g., impulsivity, stress, and cognition), leveraging idiographic methods, especially those that clarify relation directionality in order to understand individual differences and contextual factors that may moderate or underlie daily gender expression and depressive symptoms (Gates & Molenaar, 2012). They also advocate for future complementary qualitative data collection to gain insights into the adolescent-specific meaning and social experience of gender expression (Brown et al., 2023).

Third, some data collection took place during the COVID-19 pandemic. This was a unique time in the development of youth, with increases in depression (Panchal et al., 2023). Regardless, the current sample reported low-to-moderate overall depressive symptoms, with 13 individuals at risk for clinical depression (Thabrew et al., 2018). Moreover, daily fluctuations in gender expression and the strength of daily correlations were both unrelated to average levels of depressive symptoms, consistent with findings reported in an older sample (Yan et al., 2024) and importantly emphasize that gender expression matters for the mental health of some youth, but fluctuations nor congruence in general predict better or worse wellbeing. Therefore, the study conclusions about the adolescent-specific nature of daily relations between gender and symptoms concern symptom variations regardless of symptom levels, and thus, may reasonably generalize beyond clinical diagnoses and COVID-19 (although the nature of the heterogeneity may change). These are exciting areas for future work.

## Conclusion

Adolescence is a transformative developmental period – and one in which gender plays a unique role in daily life, including mental health. Leveraging 100 days of data from over 100 youth, daily relations between gender expression and depressive symptoms were examined at the average group-level, as well as at the heterogeneous individual-level. Adolescent reports of daily masculinity and femininity varied from day-to-day. Although cisgender boys – on average – showed a gender congruence effect, such that days with reduced masculinity co-occurred with heightened depressive symptoms, this effect was only apparent for about a quarter of the individual cisboys in person-specific analyses. Cisgender girls and gender-expansive youth evidenced notable heterogeneity in their daily links between gender expression and daily depressive symptoms. This variability highlights the person-specific ways in which gender expression matters for adolescent mental health and the utility in capturing the multifaceted nature of gender expression and its psychological implications.

## Supplementary information

Supplementary Materials

## Footnotes

1 Youth identified as gender non-conforming (*n* = 2), genderfluid (*n* = 1), and transgender (*n* = 2).

2 Due to statistical power, gender-expansive individuals were only included in person-specific analyses.

3 Individuals included in the person-specific analyses vary since adolescents were excluded if they showed no fluctuations (i*SD* = 0) in their daily depressive symptoms and/or daily gender expression, masculinity, or femininity.

4 The same pattern of results was found in sensitivity analyses including participants with completion rates between 50 and 79%. See Supplementary Material, Table S1.
