# Supplementary material for: Average Links Between Daily Gender Expression and Depressive Symptoms Do Not Describe Individual Adolescents
Source: J Youth Adolesc. 2025 Apr 15;54(9):2196–208. doi: 10.1007/s10964-025-02184-x (PMC12420686; doi:10.1007/s10964-025-02184-x)
Supplement: Supplementary file 1 — Supplementary Materials [file 10964_2025_2184_MOESM1_ESM.docx]

**Article Title:** Average Links Between Daily Gender Expression and Depressive Symptoms Do Not Describe Individual Adolescents

**Authors:** Ran Yan, Christel M. Portengen, Natasha Chaku, & Adriene M. Beltz

**Journal:** Journal of Youth and Adolescence

**Corresponding Author:** Adriene M. Beltz

**Supplementary Material**

| **Table S1.** Multilevel model predicting depressive symptoms by cisgender and gender expression continuum, with an age covariate (including participants with 50% or above completion rate) | | | | | | | | | | |
| --- | --- | --- | --- | --- | --- | --- | --- | --- | --- | --- |
|  |  | **Full sample**  **(N=127)** | |  | **Cisgender boys**  **(N=53)** | |  | | **Cisgender girls**  **(N=74)** | |
| **Fixed effects** |  | *b (SE)* | *p* |  | *b (SE)* | *p* | |  | *b (SE)* | *p* |
|  | Intercept | 0.022(0.054) | .681 |  | 0.962(0.039) | .017 | |  | 0.149(0.072) | .041 |
|  | Age | **0.022(0.010)** | **.037** |  | 0.009(0.005) | .083 | |  | 0.017(0.014) | .247 |
|  | Gender (0 = cisboys) | **0.108(0.045)** | **.017** |  | __ | __ | |  | __ | __ |
|  | Continuum | **-0.036(0.017)** | **.035** |  | **-0.048(0.016)** | **.006** | |  | 0.032(0.017) | .069 |
|  | Gender X Continuum | **0.071(0.022)** | **.002** |  | __ | __ | |  | __ | __ |
| **Random effects** |  |  |  |  |  |  | |  |  |  |
|  | Intercept | 0.046(0.007) | <.001 |  | 0.045(0.012) | <.001 | |  | 0.080(0.016) | <.001 |
|  | Continuum | 0.008(0.002) | <.001 |  | 0.010(0.003) | <.001 | |  | 0.012(0.004) | .002 |
| **Model fit** |  |  |  |  |  |  | |  |  |  |
|  | AIC | -6511.17 |  |  | -6797.04 |  | |  | -1553.05 |  |
| *Note*. Statistically significant fixed effects are in bold. | | | | | | | | | | |

| **Table S2.** Multilevel model predicting depressive symptoms by cisgender and masculinity, with an age covariate | | | | | | | | | |
| --- | --- | --- | --- | --- | --- | --- | --- | --- | --- |
|  |  | **Full sample**  **(N=101)** | |  | **Cisgender boys**  **(N=43)** | |  | **Cisgender girls**  **(N=58)** | |
| **Fixed effects** |  | *b (SE)* | *p* |  | *b (SE)* | *p* |  | *b (SE)* | *p* |
|  | Intercept | 0.058(0.053) | .282 |  | 0.075(0.025) | .005 |  | 0.191(0.076) | .014 |
|  | Age | 0.009(0.011) | .439 |  | 0.004(0.003) | .197 |  | -0.007(0.015) | .648 |
|  | Gender (0 = cisboys) | 0.082(0.045) | .072 |  | __ | __ |  | __ | __ |
|  | Masculinity | **-0.034(0.013)** | **.009** |  | **-0.031(0.011)** | **.006** |  | -0.001(0.014) | .944 |
|  | Gender X Masculinity | **0.042(0.017)** | **.016** |  | __ | __ |  | __ | __ |
| **Random effects** |  |  |  |  |  |  |  |  |  |
|  | Intercept | 0.040(0.006) | <.001 |  | 0.016(0.004) | <.001 |  | 0.070(0.014) | <.001 |
|  | Masculinity | 0.004(0.001) | <.001 |  | 0.004(0.001) | <.001 |  | 0.005(0.002) | .003 |
| **Model fit** |  |  |  |  |  |  |  |  |  |
|  | AIC | -6345.75 |  |  | -6115.33 |  |  | -1774.20 |  |
| *Note*. Statistically significant fixed effects are in bold. | | | | | | | | | |

| **Table S3.** Multilevel model predicting depressive symptoms by cisgender and femininity, with an age covariate | | | |
| --- | --- | --- | --- |
|  |  | **Full sample (N=101)** | |
| **Fixed effects** |  | *b(SE)* | *p* |
|  | Intercept | 0.001(0.480) | .977 |
|  | Age | 0.010(0.010) | .284 |
|  | Gender (0=cisboys) | **0.137(0.041)** | **.003** |
|  | Femininity | 0.001(0.011) | .964 |
|  | Gender X Femininity | -0.017(0.015) | .254 |
| **Random effects** |  |  |  |
|  | Intercept | 0.035(0.005) | <.001 |
|  | Femininity | 0.002(0.001) | <.001 |
| **Model fit** |  |  |  |
|  | AIC | -6232.88 |  |
| *Note*. Statistically significant fixed effects are in bold. | | | |


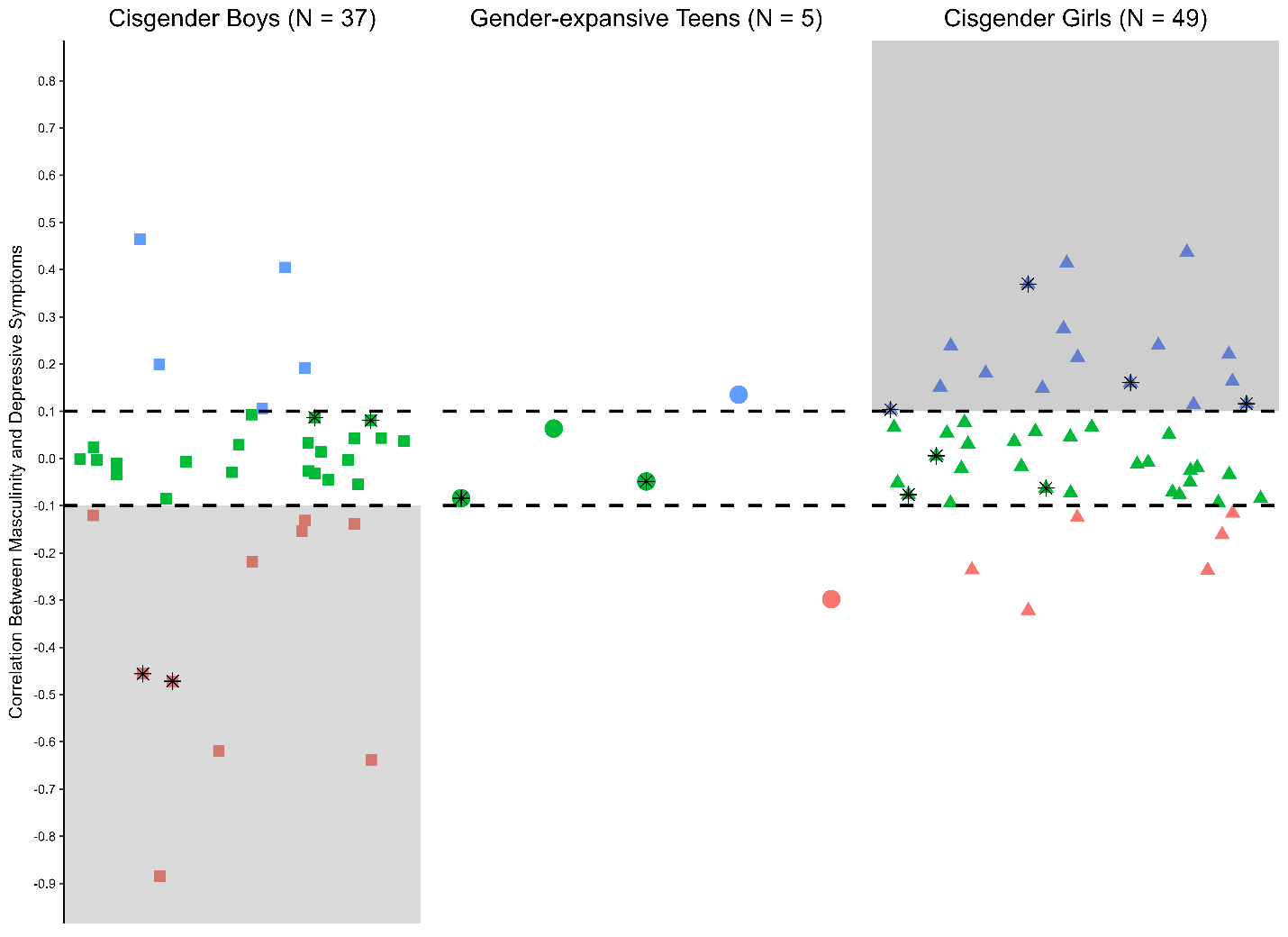


**Figure S1.** Scatterplot displaying adolescent-specific correlations between daily masculinity and depressive symptoms among 91 adolescents (86 cisgender adolescents + 5 gender-expansive adolescents). The y-axis shows the strength and direction of the correlations, and the x-axis represents individual adolescents. Cisgender boys are indicated by solid squares and are placed on the left side of the plot, gender-expansive teens are indicated by solid circles and are in the middle, and cisgender girls are indicated by solid triangles and appear on the right. Youth at risk for clinical depression (*n*=13) are overlaid with a black asterisk. The thick dashed lines at *r*=0.10 and *r*=-0.10 demarcate the threshold for the smallest effect size of interest. Positive correlations, depicted in blue and positioned above the dashed lines, and negative correlations, shown in red and located below the lines, signify meaningful correlations at the individual level. The gray shaded areas highlight the areas of gender congruence effects.


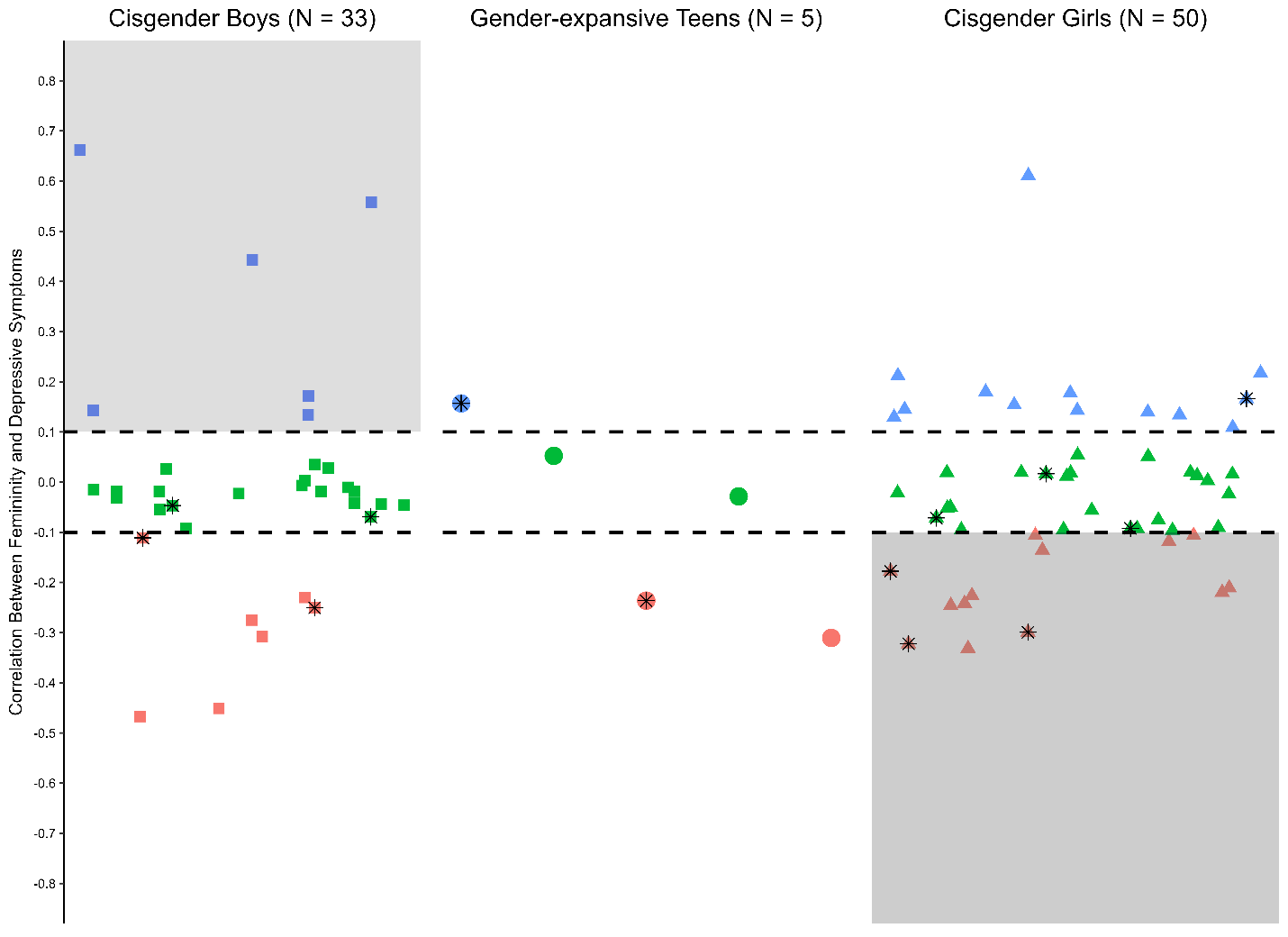


**Figure S2.** Scatterplot displaying adolescent-specific correlations between daily femininity and depressive symptoms among 88 adolescents (83 cisgender adolescents + 5 gender-expansive individuals). The y-axis shows the strength and direction of the correlations, and the x-axis represents individual adolescents. Cisgender boys are indicated by solid squares and are placed on the left side of the plot, gender-expansive teens are indicated by solid circles and are in the middle, and cisgender girls are indicated by solid triangles and appear on the right. Youth at risk for clinical depression (*n*=13) are overlaid with a black asterisk. The thick dashed lines at *r*=0.10 and *r*=-0.10 demarcate the threshold for the smallest effect size of interest. Positive correlations, depicted in blue and positioned above the dashed lines, and negative correlations, shown in red and located below the lines, signify meaningful correlations at the individual level. The gray shaded areas highlight the areas of gender congruence effects.

# Fluctuations in Daily Gender Expression and Depressive Symptoms across 100 Days:

**Sensitivity Analyses Accounting for Family-level Nesting**

Accounting for family-level nesting, a multilevel, intercept-only model was conducted with gender expression fluctuations (i*SD*s) as the outcome. Average gender expression i*SD*s differed from zero, indicating significant fluctuations, *b=*0.27, *SE=*0.02, *t*=14.66, *p*<.001. In addition, gender was significantly associated with daily gender expression (i*M*s), *b=*2.44, *SE=*0.14, *t*=17.10, *p*<.001, such that cisgender boys scored higher on the continuum (i.e., higher masculinity and lower femininity) than cisgender girls. Gender was not significantly linked to fluctuations in daily gender expression (i*SD*s), *b=*-0.02, *SE=*0.03, *t*=-0.66, *p*=.509. There was a significant association between fluctuations (i*SD*) in daily gender expression and average daily depressive symptoms (i*M*s), *b=*0.19, *SE=*0.08, *t*=2.28, *p*=.025, but not age, *b=*-0.01, *SE=*0.01, *t*=-0.92, *p*=.359*.*

Regarding masculinity, fluctuations significantly differed from 0, *b=*0.32, *SE=*0.03, *t*=12.89, *p*<.001. Gender was significantly associated with average daily masculinity (i*M*s), *b=*2.38, *SE=*0.18, *t*=13.11, *p*<.001, such that cisgender boys reported higher levels of masculinity compared to cisgender girls. Fluctuations in daily masculinity (i*SD*s) were not significantly linked to gender, *b=*0.03, *SE=*0.05, *t*=0.70, *p*=.489, but were significantly associated with age, *b=*-0.03, *SE=*0.01, *t*=-2.09, *p*=.039.

Regarding femininity, fluctuations significantly differed from 0, *b=*0.03, *SE=*0.03, *t*=13.19, *p*<.001. Gender was significantly associated with femininity (i*M*s), *b=*-2.51, *SE=*0.15, *t*=-16.64, *p*<.001, such that cisgender boys reported lower levels of femininity than cisgender girls. Fluctuations in daily femininity (i*SD*s) were not significantly linked to gender, *b=*-0.06, *SE=*0.05, *t*=-1.21, *p*=.229, or age, *b=*-0.01, *SE=*0.01, *t*=-0.35, *p*=.729.

Individual-level Links between Daily Gender Expression and Depressive Symptoms in Cisgender Adolescents: Sensitivity Analyses for Gender Congruence Comparisons

When limiting analyses to one sibling per family, there was no significant difference in depressive symptoms (i*M*s) between adolescents who showed a gender congruence effect (*n*=20; *M*=0.18, *SD*=0.33) and those who did not (*n*=17; *M*=0.10, *SD*=0.12), *t*(35)=0.99, *p*=.330. Similarly, age did not differ significantly between these two groups (*M*=13.66, *SD*=1.94 vs. *M*=13.76, *SD*=1.49, respectively), *t*(35)=-0.17, *p*=.865.

**Individual-level Links between Gender Expression Continuum and Depressive Symptoms in Gender-expansive Adolescents: Description of Person-Specific Correlations**

Person-specific correlations for the 5 gender-expansive adolescents are calculated. Average gender expression (i*M*) ranged from 1.58 to 4.77, covering almost the full continuum. Average gender expression fluctuations (i*SD*) ranged from 0.21 to 0.87, capturing the stability and fluidity in gender expression. The feminine-to-masculine continuum provided reliable information regarding the daily gender experience for gender-expansive adolescents. For example, participants’ gender expression scores fell within expected range of their *identified* gender and those who did not adopt a binary gender identity showed large day-to-day fluctuations (i*SD*s) in the feminine-to-masculine continuum. As expected, individual differences in the relation between daily gender expression and depressive symptoms extended to gender-expansive adolescents.
